# Poly[[diaqua­tris­(μ_2_-3-methyl­pyridine-2-carboxyl­ato)(3-methyl­pyridine-2-car­boxyl­ato)sodiumterbium(III)] ethanol monosolvate monohydrate]

**DOI:** 10.1107/S1600536810038456

**Published:** 2010-10-02

**Authors:** Taewoo Lee, Sung Kwon Kang

**Affiliations:** a Department of Chemistry, Chungnam National University, Daejeon 305-764, Republic of Korea

## Abstract

In the title compound, {[NaTb(C_7_H_6_NO_2_)_4_(H_2_O)_2_]·C_2_H_5_OH·H_2_O}_*n*_, the Tb^III^ atom is eight-coordinated in a slightly distorted square-anti­prismatic geometry defined by four carboxyl­ate O atoms and four pyridine N atoms. The bond lengths lie within the range 2.3000 (2)–2.326 (2) Å for the Tb—O bonds and 2.543 (3)–2.553 (3) Å for the Tb—N bonds. The Na^I^ atom is five-coordinated by two water O atoms and three carboxyl­ate O atoms in a distorted square-pyramidal geometry. In the crystal, inter­molecular O—H⋯O hydrogen bonds link the mol­ecules into a three-dimensional network.

## Related literature

For general background to luminescent compounds, see: Fan *et al.* (2009 ▶); Oh *et al.* (2010 ▶); Seo *et al.* (2010 ▶); Zhou *et al.* (2010 ▶). For luminescence properties of metal compounds, see: Godlewska *et al.* (2008 ▶); Kang (2010 ▶); Kim *et al.* (2010 ▶); Legendziewicz (2002 ▶); Lis *et al.* (2009 ▶); Seo *et al.* (2009 ▶). 
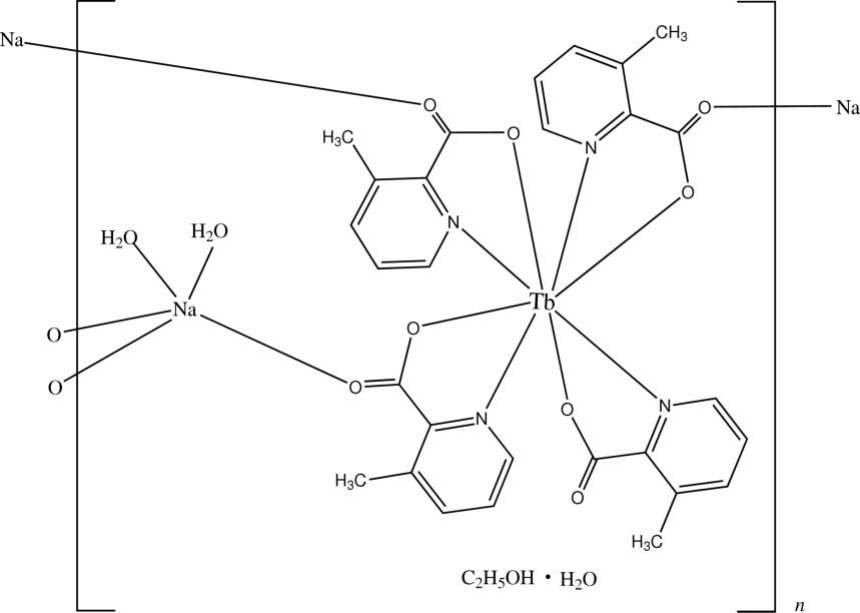


## Experimental

### 

#### Crystal data

[NaTb(C_7_H_6_NO_2_)_4_(H_2_O)_2_]·C_2_H_6_O·H_2_O
*M* *_r_* = 826.54
Orthorhombic, 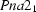

*a* = 18.4662 (6) Å
*b* = 18.5290 (5) Å
*c* = 9.8939 (3) Å
*V* = 3385.30 (18) Å^3^
*Z* = 4
Mo *K*α radiation
μ = 2.17 mm^−1^
*T* = 174 K
0.16 × 0.11 × 0.07 mm

#### Data collection

Bruker SMART CCD area-detector diffractometer
Absorption correction: multi-scan (*SADABS*; Bruker, 2002 ▶) *T*_min_ = 0.704, *T*_max_ = 0.856
44141 measured reflections
6652 independent reflections
5995 reflections with *I* > 2σ(*I*)
*R*_int_ = 0.038

#### Refinement

*R*[*F* ^2^ > 2σ(*F* ^2^)] = 0.022
*wR*(*F* ^2^) = 0.045
*S* = 1.07
6652 reflections
460 parameters
6 restraints
H atoms treated by a mixture of independent and constrained refinement
Δρ_max_ = 0.51 e Å^−3^
Δρ_min_ = −0.56 e Å^−3^
Absolute structure: Flack (1983 ▶), 2234 Friedel pairs
Flack parameter: 0.001 (6)

### 

Data collection: *SMART* (Bruker, 2002 ▶); cell refinement: *SAINT* (Bruker, 2002 ▶); data reduction: *SAINT*; program(s) used to solve structure: *SHELXS97* (Sheldrick, 2008 ▶); program(s) used to refine structure: *SHELXL97* (Sheldrick, 2008 ▶); molecular graphics: *DIAMOND* (Brandenburg, 2010 ▶); software used to prepare material for publication: *WinGX* (Farrugia, 1999 ▶).

## Supplementary Material

Crystal structure: contains datablocks global, I. DOI: 10.1107/S1600536810038456/is2603sup1.cif

Structure factors: contains datablocks I. DOI: 10.1107/S1600536810038456/is2603Isup2.hkl

Additional supplementary materials:  crystallographic information; 3D view; checkCIF report

## Figures and Tables

**Table 1:** Selected bond lengths (Å)

Na41—O9	2.324 (2)
Na41—O29^i^	2.390 (3)
Na41—O39^ii^	2.374 (2)
Na41—O42	2.265 (3)
Na41—O43	2.476 (3)

Symmetry codes: (i) 
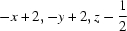
; (ii) 
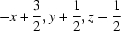
.

**Table 2:** Hydrogen-bond geometry (Å, °)

*D*—H⋯*A*	*D*—H	H⋯*A*	*D*⋯*A*	*D*—H⋯*A*
O42—H42*A*⋯O47	0.81 (2)	1.95 (2)	2.742 (4)	163 (4)
O42—H42*B*⋯O38^ii^	0.81 (2)	2.01 (2)	2.819 (3)	169 (4)
O43—H43*A*⋯O28^i^	0.82 (2)	1.99 (2)	2.796 (3)	171 (4)
O43—H43*A*⋯O29^i^	0.82 (2)	2.53 (3)	3.050 (3)	122 (3)
O43—H43*B*⋯O44^i^	0.81 (2)	2.02 (2)	2.794 (4)	162 (3)
O44—H44*A*⋯O8	0.80 (4)	2.31 (4)	3.103 (4)	170 (4)
O44—H44*A*⋯O9	0.80 (4)	2.34 (4)	2.943 (4)	133 (4)
O44—H44*B*⋯O43	0.80 (4)	2.07 (4)	2.790 (4)	151 (4)
O47—H47⋯O19^ii^	0.80 (2)	1.96 (2)	2.726 (3)	159 (4)

Symmetry codes: (i) 
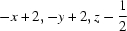
; (ii) 
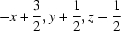
.

**Fig. 1.**
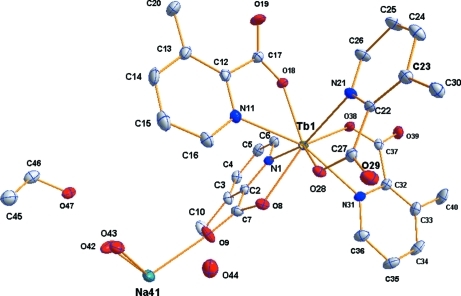
Molecular structure of the title complex showing the atom-numbering scheme and 50% probability ellipsoids. H atoms have been omitted for clarity.

## supplementary crystallographic information

### Comment

Luminescent metal compounds with N-containing ligands have been reported in the investigation of their interesting photophysical properties and various coordination modes (Seo *et al.*, 2010; Zhou *et al.*, 2010; Fan *et al.*, 2009). Especially, lanthanide metal complexes have been extensively studied due to their unique luminescence properties (Lis *et al.*, 2009; Godlewska *et al.*, 2008; Legendziewicz, 2002). As an extension of our work (Kang, 2010; Oh *et al.*, 2010; Kim *et al.*, 2010; Seo *et al.*, 2009) on luminescent complexes, herein, we report the crystal structure and luminescent properties of the title Tb(III) chloride complex with 3-methylpyridine-2-carboxylic acid (3-methylpicolinic acid), (I).

In the title compound, {[NaTb(H_2_O)_2_(C_7_H_6_NO_2_)_4_].C_2_H_5_OH.H_2_O}*_n_*, the Tb^III^ atom is eight-coordinated within a slightly distorted square antiprismatic geometry. The Tb^III^ atom is coordinated to the four carboxylate-O atoms and four pyridine-N atoms. The Tb—O bond distances are within the range of 2.300 (2)–2.326 (2) Å (Table 1), which are significantly shorter than the sum of the covalent radii of Tb and O atoms (2.420 Å). The Na^+^ ion is five-coordinated with two water-O atoms and three carboxylate-O atoms to form a distorted square pyramidal geometry. In the crystal structure, intermolecular O—H···O hydrogen bonds (Table 2) link the uncoordinated water molecule to the coordinated picolinic ligands and further link the molecules into a three-dimensional network.

The title compound exhibits an intense emission at 543 nm upon 326 nm excitation in PL spectra with 325 nm of He—Cd laser excitation wavelength

### Experimental

Terbium trichloride solution was prepared by dissolving TbCl_3_.6H_2_O (0.27 g, 1.0 mmol; Aldrich) in absolute ethanol (20 ml) at room temperature with stirring. The ligand solution was prepared by dissolving 3-methylpicolinic acid (0.55 g, 4.0 mmol; Aldrich) in absolute ethanol (30 ml) at room temperature with stirring. The pH of the ligand solution was adjusted to about 5.1 with 2 N NaOH solution. The Terbium trichloride solution was added dropwise slowly to the ligand solution. The reaction mixture was stirred for 1 h at room temperature. Colourless crystals of (I) were obtained at room temperature over a period of a few weeks. The complex was recrystallized from the mixture of distilled water and absolute ethanol solution.

### Refinement

The O—H atoms were located in a difference Fourier map and refined with O—H = 0.81±0.01 Å. The remaining H atoms were positioned geometrically and refined using a riding model, with C—H = 0.93–0.97 Å, and with *U*_iso_(H) = 1.2*U*_eq_ (C) for aromatic and methylene-H and 1.5*U*_eq_(C) for methyl-H atoms. The maximum and minimum residual electron density peaks of 0.51 and -0.56 e Å^-3^, respectively, were located 0.85 Å and 0.57 Å from the Tb1 atom, respectively.

### Crystal data

**Table:**

[NaTb(C_7_H_6_NO_2_)_4_(H_2_O)_2_]·C_2_H_6_O·H_2_O	*F*(000) = 1664
*M**_r_* = 826.54	*D*_x_ = 1.622 Mg m^−^^3^
Orthorhombic, *P**n**a*2_1_	Mo *K*α radiation, λ = 0.71073 Å
Hall symbol: P 2c -2n	Cell parameters from 9041 reflections
*a* = 18.4662 (6) Å	θ = 2.3–28.2°
*b* = 18.5290 (5) Å	µ = 2.17 mm^−^^1^
*c* = 9.8939 (3) Å	*T* = 174 K
*V* = 3385.30 (18) Å^3^	Block, colourless
*Z* = 4	0.16 × 0.11 × 0.07 mm

### Data collection

**Table:**

Bruker SMART CCD area-detector diffractometer	5995 reflections with *I* > 2σ(*I*)
φ and ω scans	*R*_int_ = 0.038
Absorption correction: multi-scan (*SADABS*; Bruker, 2002)	θ_max_ = 28.3°, θ_min_ = 1.6°
*T*_min_ = 0.704, *T*_max_ = 0.856	*h* = −24→20
44141 measured reflections	*k* = −18→24
6652 independent reflections	*l* = −13→8

### Refinement

**Table:**

Refinement on *F*^2^	H atoms treated by a mixture of independent and constrained refinement
Least-squares matrix: full	*w* = 1/[σ^2^(*F*_o_^2^) + (0.0185*P*)^2^ + 0.0181*P*] where *P* = (*F*_o_^2^ + 2*F*_c_^2^)/3
*R*[*F*^2^ > 2σ(*F*^2^)] = 0.022	(Δ/σ)_max_ = 0.004
*wR*(*F*^2^) = 0.045	Δρ_max_ = 0.51 e Å^−^^3^
*S* = 1.07	Δρ_min_ = −0.56 e Å^−^^3^
6652 reflections	Absolute structure: Flack (1983), 2234 Friedel pairs
460 parameters	Flack parameter: 0.001 (6)
6 restraints	

### Special details

**Table:**

Geometry. All e.s.d.'s (except the e.s.d. in the dihedral angle between two l.s. planes) are estimated using the full covariance matrix. The cell e.s.d.'s are taken into account individually in the estimation of e.s.d.'s in distances, angles and torsion angles; correlations between e.s.d.'s in cell parameters are only used when they are defined by crystal symmetry. An approximate (isotropic) treatment of cell e.s.d.'s is used for estimating e.s.d.'s involving l.s. planes.

### Fractional atomic coordinates and isotropic or equivalent isotropic displacement parameters (Å^2^)

**Table:**

	*x*	*y*	*z*	*U*_iso_*/*U*_eq_	
Tb1	0.878991 (6)	0.769501 (6)	0.19856 (3)	0.01726 (4)	
N1	0.75484 (13)	0.80674 (13)	0.1090 (2)	0.0206 (5)	
C2	0.74647 (16)	0.87607 (16)	0.0698 (3)	0.0218 (6)	
C3	0.68063 (18)	0.90306 (18)	0.0214 (3)	0.0302 (8)	
C4	0.62409 (18)	0.8540 (2)	0.0116 (4)	0.0331 (9)	
H4	0.5794	0.8695	−0.0209	0.04*	
C5	0.63268 (17)	0.7836 (2)	0.0487 (3)	0.0304 (8)	
H5	0.5945	0.751	0.0412	0.036*	
C6	0.69877 (17)	0.76174 (18)	0.0972 (3)	0.0264 (7)	
H6	0.7047	0.7138	0.1227	0.032*	
C7	0.81601 (17)	0.91984 (16)	0.0816 (3)	0.0262 (7)	
O8	0.86652 (10)	0.89129 (11)	0.1516 (2)	0.0254 (5)	
O9	0.82067 (13)	0.97871 (12)	0.0241 (3)	0.0380 (6)	
C10	0.6682 (2)	0.9806 (2)	−0.0189 (5)	0.0563 (12)	
H10A	0.6886	0.989	−0.1068	0.084*	
H10B	0.6911	1.0119	0.0457	0.084*	
H10C	0.6172	0.9904	−0.0211	0.084*	
N11	0.94179 (14)	0.77843 (13)	−0.0307 (2)	0.0242 (6)	
C12	0.93444 (17)	0.72055 (16)	−0.1136 (3)	0.0240 (7)	
C13	0.9646 (2)	0.71841 (19)	−0.2442 (3)	0.0331 (9)	
C14	1.0026 (2)	0.77989 (19)	−0.2830 (4)	0.0407 (10)	
H14	1.0233	0.7813	−0.3687	0.049*	
C15	1.01063 (19)	0.8380 (2)	−0.2005 (4)	0.0404 (9)	
H15	1.0366	0.8783	−0.2285	0.049*	
C16	0.97899 (18)	0.83566 (18)	−0.0733 (3)	0.0316 (8)	
H16	0.9839	0.8752	−0.0161	0.038*	
C17	0.89107 (17)	0.65991 (18)	−0.0524 (3)	0.0231 (7)	
O18	0.85345 (12)	0.67662 (11)	0.0515 (2)	0.0233 (5)	
O19	0.89462 (13)	0.59921 (13)	−0.1012 (2)	0.0421 (6)	
C20	0.9552 (2)	0.6564 (2)	−0.3396 (3)	0.0529 (12)	
H20A	0.9923	0.6211	−0.3232	0.079*	
H20B	0.9588	0.6734	−0.431	0.079*	
H20C	0.9085	0.6349	−0.3257	0.079*	
N21	0.97062 (13)	0.67355 (13)	0.2657 (2)	0.0218 (6)	
C22	1.03261 (16)	0.69539 (16)	0.3236 (3)	0.0194 (6)	
C23	1.08422 (19)	0.64699 (18)	0.3738 (3)	0.0252 (8)	
C24	1.06794 (18)	0.57326 (18)	0.3599 (3)	0.0321 (8)	
H24	1.1007	0.5391	0.3917	0.039*	
C25	1.00540 (19)	0.55083 (17)	0.3009 (4)	0.0355 (9)	
H25	0.9954	0.5019	0.2915	0.043*	
C26	0.95664 (18)	0.60265 (16)	0.2549 (3)	0.0311 (8)	
H26	0.9133	0.5878	0.2157	0.037*	
C27	1.04130 (17)	0.77676 (15)	0.3275 (3)	0.0211 (7)	
O28	0.99116 (11)	0.81287 (11)	0.2681 (2)	0.0216 (5)	
O29	1.09365 (13)	0.80420 (12)	0.3846 (2)	0.0337 (5)	
C30	1.1533 (2)	0.66829 (19)	0.4411 (4)	0.0365 (9)	
H30A	1.1797	0.7007	0.3835	0.055*	
H30B	1.182	0.626	0.4579	0.055*	
H30C	1.1428	0.6919	0.5253	0.055*	
N31	0.85257 (14)	0.81025 (13)	0.4399 (2)	0.0214 (5)	
C32	0.81292 (16)	0.76643 (16)	0.5184 (3)	0.0209 (7)	
C33	0.79403 (18)	0.78474 (18)	0.6512 (3)	0.0280 (8)	
C34	0.81700 (15)	0.85172 (17)	0.6973 (5)	0.0374 (7)	
H34	0.8048	0.8663	0.7843	0.045*	
C35	0.85745 (19)	0.8969 (2)	0.6171 (3)	0.0376 (9)	
H35	0.8725	0.9417	0.6486	0.045*	
C36	0.87514 (16)	0.87394 (18)	0.4880 (4)	0.0298 (8)	
H36	0.9034	0.9035	0.4334	0.036*	
C37	0.78728 (16)	0.69799 (17)	0.4476 (3)	0.0213 (7)	
O38	0.80812 (12)	0.69262 (11)	0.3243 (2)	0.0254 (5)	
O39	0.74967 (12)	0.65377 (12)	0.5063 (2)	0.0306 (5)	
C40	0.7506 (2)	0.7367 (2)	0.7431 (3)	0.0462 (11)	
H40A	0.748	0.7579	0.8315	0.069*	
H40B	0.7734	0.6902	0.7491	0.069*	
H40C	0.7025	0.7312	0.7075	0.069*	
Na41	0.84271 (7)	1.08389 (6)	−0.09718 (12)	0.0286 (3)	
O42	0.77005 (16)	1.07464 (14)	−0.2808 (3)	0.0468 (7)	
H42A	0.764 (2)	1.058 (2)	−0.356 (3)	0.056*	
H42B	0.7432 (18)	1.1072 (16)	−0.257 (3)	0.056*	
O43	0.96139 (14)	1.04567 (12)	−0.1891 (2)	0.0340 (6)	
H43A	0.9724 (19)	1.0870 (11)	−0.209 (3)	0.041*	
H43B	0.9744 (18)	1.0202 (16)	−0.250 (3)	0.041*	
O44	0.97142 (17)	1.01756 (16)	0.0874 (3)	0.0452 (7)	
H44A	0.941 (2)	0.988 (2)	0.098 (4)	0.052 (14)*	
H44B	0.984 (2)	1.023 (2)	0.011 (4)	0.062 (15)*	
C45	0.8279 (2)	1.0581 (3)	−0.6322 (5)	0.0689 (13)	
H45A	0.854	1.0534	−0.7156	0.103*	
H45B	0.8577	1.0418	−0.5588	0.103*	
H45C	0.8152	1.1078	−0.6182	0.103*	
C46	0.7615 (2)	1.0140 (2)	−0.6381 (4)	0.0559 (12)	
H46A	0.7321	1.0304	−0.7134	0.067*	
H46B	0.7748	0.9642	−0.6553	0.067*	
O47	0.71951 (15)	1.01711 (15)	−0.5181 (3)	0.0495 (7)	
H47	0.6882 (18)	1.0467 (18)	−0.525 (4)	0.059*	

### Atomic displacement parameters (Å^2^)

**Table:**

	*U*^11^	*U*^22^	*U*^33^	*U*^12^	*U*^13^	*U*^23^
Tb1	0.01829 (7)	0.01596 (6)	0.01754 (6)	−0.00143 (5)	0.00021 (9)	−0.00065 (11)
N1	0.0205 (14)	0.0203 (14)	0.0209 (12)	−0.0017 (11)	0.0016 (10)	−0.0017 (11)
C2	0.0209 (17)	0.0235 (16)	0.0210 (14)	0.0002 (13)	0.0029 (13)	0.0008 (13)
C3	0.0251 (19)	0.035 (2)	0.0305 (16)	0.0036 (16)	−0.0028 (14)	0.0067 (15)
C4	0.025 (2)	0.044 (2)	0.0305 (19)	0.0014 (18)	−0.0022 (16)	0.0036 (17)
C5	0.020 (2)	0.044 (2)	0.0280 (17)	−0.0083 (15)	0.0015 (14)	−0.0023 (15)
C6	0.026 (2)	0.0266 (17)	0.0266 (16)	−0.0051 (15)	0.0025 (14)	0.0011 (14)
C7	0.0258 (19)	0.0212 (17)	0.0316 (17)	−0.0033 (14)	0.0038 (14)	−0.0019 (14)
O8	0.0195 (12)	0.0218 (11)	0.0349 (12)	−0.0002 (9)	−0.0038 (9)	−0.0015 (9)
O9	0.0319 (14)	0.0284 (13)	0.0535 (15)	−0.0011 (11)	0.0010 (12)	0.0213 (12)
C10	0.036 (2)	0.048 (3)	0.085 (3)	0.006 (2)	−0.017 (2)	0.022 (2)
N11	0.0229 (16)	0.0250 (15)	0.0247 (13)	−0.0018 (12)	0.0002 (11)	0.0026 (11)
C12	0.0243 (19)	0.0261 (18)	0.0215 (15)	0.0066 (13)	0.0013 (13)	0.0045 (14)
C13	0.038 (2)	0.040 (2)	0.0216 (16)	0.0124 (17)	0.0079 (15)	0.0019 (15)
C14	0.047 (2)	0.049 (2)	0.025 (3)	0.0076 (16)	0.0158 (18)	0.0132 (18)
C15	0.034 (2)	0.044 (2)	0.044 (2)	0.0016 (17)	0.0119 (17)	0.0187 (19)
C16	0.030 (2)	0.0296 (18)	0.0351 (18)	−0.0032 (15)	0.0018 (15)	0.0073 (15)
C17	0.0228 (18)	0.0253 (17)	0.0213 (15)	0.0001 (13)	−0.0007 (13)	−0.0013 (13)
O18	0.0246 (12)	0.0231 (11)	0.0220 (10)	−0.0013 (10)	0.0031 (9)	−0.0037 (9)
O19	0.0596 (17)	0.0266 (13)	0.0401 (14)	−0.0064 (12)	0.0216 (13)	−0.0127 (12)
C20	0.085 (3)	0.047 (2)	0.026 (2)	0.017 (2)	0.0180 (18)	−0.0006 (16)
N21	0.0210 (15)	0.0175 (13)	0.0268 (13)	−0.0006 (11)	0.0006 (11)	0.0017 (10)
C22	0.0241 (18)	0.0160 (15)	0.0180 (13)	−0.0017 (13)	0.0026 (12)	0.0008 (12)
C23	0.025 (2)	0.0234 (17)	0.0274 (17)	0.0017 (15)	0.0017 (15)	0.0037 (14)
C24	0.0251 (19)	0.0253 (18)	0.046 (2)	0.0061 (15)	0.0007 (16)	0.0093 (16)
C25	0.037 (2)	0.0160 (17)	0.054 (2)	0.0014 (15)	−0.0021 (18)	0.0023 (16)
C26	0.030 (2)	0.0186 (16)	0.0453 (19)	−0.0021 (14)	−0.0054 (15)	0.0001 (14)
C27	0.0213 (18)	0.0237 (17)	0.0184 (14)	−0.0023 (13)	0.0035 (12)	0.0010 (13)
O28	0.0177 (11)	0.0172 (11)	0.0298 (11)	0.0004 (9)	−0.0021 (9)	0.0012 (9)
O29	0.0307 (14)	0.0242 (12)	0.0463 (14)	−0.0051 (11)	−0.0151 (12)	−0.0019 (11)
C30	0.034 (2)	0.032 (2)	0.043 (2)	0.0024 (17)	−0.0071 (17)	0.0057 (17)
N31	0.0195 (14)	0.0220 (14)	0.0227 (12)	0.0004 (12)	0.0016 (11)	−0.0036 (11)
C32	0.0148 (17)	0.0289 (17)	0.0192 (14)	−0.0007 (14)	0.0005 (12)	−0.0018 (13)
C33	0.0235 (19)	0.039 (2)	0.0215 (14)	−0.0012 (15)	−0.0014 (12)	0.0010 (13)
C34	0.0322 (17)	0.056 (2)	0.0234 (13)	−0.0029 (15)	0.003 (2)	−0.015 (3)
C35	0.039 (2)	0.036 (2)	0.038 (2)	−0.0087 (17)	0.0002 (17)	−0.0173 (17)
C36	0.026 (2)	0.0308 (18)	0.0329 (18)	−0.0072 (15)	0.0039 (14)	−0.0073 (15)
C37	0.0177 (17)	0.0253 (16)	0.0210 (14)	0.0039 (14)	−0.0012 (12)	0.0026 (13)
O38	0.0324 (14)	0.0240 (12)	0.0199 (10)	−0.0061 (10)	0.0037 (10)	−0.0026 (9)
O39	0.0327 (14)	0.0345 (13)	0.0246 (11)	−0.0096 (11)	0.0041 (10)	0.0056 (10)
C40	0.051 (3)	0.067 (3)	0.0206 (16)	−0.005 (2)	0.0059 (16)	−0.0044 (17)
Na41	0.0312 (8)	0.0212 (6)	0.0333 (7)	0.0014 (6)	0.0016 (6)	0.0032 (6)
O42	0.0501 (16)	0.0436 (16)	0.047 (2)	0.0212 (12)	−0.0120 (14)	−0.0133 (14)
O43	0.0413 (15)	0.0226 (13)	0.0382 (14)	−0.0012 (12)	0.0097 (12)	−0.0011 (11)
O44	0.0456 (19)	0.0522 (19)	0.0379 (16)	−0.0228 (15)	−0.0070 (14)	0.0054 (14)
C45	0.065 (3)	0.067 (3)	0.075 (3)	−0.007 (3)	0.012 (3)	−0.009 (3)
C46	0.052 (3)	0.063 (3)	0.053 (3)	0.006 (2)	0.001 (2)	−0.011 (2)
O47	0.052 (2)	0.0492 (18)	0.0470 (16)	0.0097 (14)	−0.0055 (14)	−0.0090 (14)

### Geometric parameters (Å, °)

**Table:**

Tb1—O8	2.315 (2)	N21—C26	1.343 (4)
Tb1—O18	2.302 (2)	C22—C23	1.400 (4)
Tb1—O28	2.326 (2)	C22—C27	1.517 (4)
Tb1—O38	2.300 (2)	C23—C24	1.406 (4)
Tb1—N1	2.553 (2)	C23—C30	1.493 (5)
Tb1—N11	2.553 (3)	C24—C25	1.359 (4)
Tb1—N21	2.543 (2)	C24—H24	0.93
Tb1—N31	2.551 (2)	C25—C26	1.393 (4)
Na41—O9	2.324 (2)	C25—H25	0.93
Na41—O29^i^	2.390 (3)	C26—H26	0.93
Na41—O39^ii^	2.374 (2)	C27—O29	1.230 (4)
Na41—O42	2.265 (3)	C27—O28	1.285 (3)
Na41—O43	2.476 (3)	C30—H30A	0.96
N1—C6	1.334 (4)	C30—H30B	0.96
N1—C2	1.351 (4)	C30—H30C	0.96
C2—C3	1.399 (4)	N31—C36	1.339 (4)
C2—C7	1.523 (4)	N31—C32	1.341 (4)
C3—C4	1.387 (4)	C32—C33	1.401 (4)
C3—C10	1.509 (5)	C32—C37	1.524 (4)
C4—C5	1.366 (5)	C33—C34	1.389 (4)
C4—H4	0.93	C33—C40	1.505 (5)
C5—C6	1.372 (4)	C34—C35	1.374 (5)
C5—H5	0.93	C34—H34	0.93
C6—H6	0.93	C35—C36	1.385 (5)
C7—O9	1.233 (3)	C35—H35	0.93
C7—O8	1.277 (4)	C36—H36	0.93
C10—H10A	0.96	C37—O39	1.221 (3)
C10—H10B	0.96	C37—O38	1.283 (3)
C10—H10C	0.96	C40—H40A	0.96
N11—C16	1.332 (4)	C40—H40B	0.96
N11—C12	1.357 (3)	C40—H40C	0.96
C12—C13	1.408 (4)	O42—H42A	0.814 (18)
C12—C17	1.507 (4)	O42—H42B	0.814 (18)
C13—C14	1.392 (5)	O43—H43A	0.817 (18)
C13—C20	1.497 (5)	O43—H43B	0.806 (17)
C14—C15	1.360 (5)	O44—H44A	0.80 (4)
C14—H14	0.93	O44—H44B	0.80 (4)
C15—C16	1.388 (4)	C45—C46	1.474 (5)
C15—H15	0.93	C45—H45A	0.96
C16—H16	0.93	C45—H45B	0.96
C17—O19	1.226 (4)	C45—H45C	0.96
C17—O18	1.279 (3)	C46—O47	1.419 (5)
C20—H20A	0.96	C46—H46A	0.97
C20—H20B	0.96	C46—H46B	0.97
C20—H20C	0.96	O47—H47	0.800 (18)
N21—C22	1.343 (4)		
			
O38—Tb1—O18	76.25 (7)	C22—N21—C26	119.5 (3)
O38—Tb1—O8	130.96 (7)	C22—N21—Tb1	117.91 (18)
O18—Tb1—O8	125.56 (7)	C26—N21—Tb1	122.4 (2)
O38—Tb1—O28	124.11 (7)	N21—C22—C23	122.6 (3)
O18—Tb1—O28	128.87 (7)	N21—C22—C27	113.6 (2)
O8—Tb1—O28	79.11 (7)	C23—C22—C27	123.8 (3)
O38—Tb1—N21	78.72 (8)	C22—C23—C24	116.2 (3)
O18—Tb1—N21	77.21 (8)	C22—C23—C30	124.8 (3)
O8—Tb1—N21	143.13 (7)	C24—C23—C30	118.9 (3)
O28—Tb1—N21	64.64 (7)	C25—C24—C23	121.4 (3)
O38—Tb1—N31	64.42 (7)	C25—C24—H24	119.3
O18—Tb1—N31	140.66 (8)	C23—C24—H24	119.3
O8—Tb1—N31	83.12 (8)	C24—C25—C26	118.6 (3)
O28—Tb1—N31	77.95 (8)	C24—C25—H25	120.7
N21—Tb1—N31	95.15 (8)	C26—C25—H25	120.7
O38—Tb1—N1	81.03 (8)	N21—C26—C25	121.6 (3)
O18—Tb1—N1	78.40 (8)	N21—C26—H26	119.2
O8—Tb1—N1	65.02 (7)	C25—C26—H26	119.2
O28—Tb1—N1	144.00 (7)	O29—C27—O28	124.2 (3)
N21—Tb1—N1	151.31 (8)	O29—C27—C22	120.4 (3)
N31—Tb1—N1	94.19 (8)	O28—C27—C22	115.5 (3)
O38—Tb1—N11	141.19 (7)	C27—O28—Tb1	126.68 (18)
O18—Tb1—N11	65.17 (7)	C27—O29—Na41^iii^	141.3 (2)
O8—Tb1—N11	78.69 (7)	C23—C30—H30A	109.5
O28—Tb1—N11	80.56 (8)	C23—C30—H30B	109.5
N21—Tb1—N11	88.57 (8)	H30A—C30—H30B	109.5
N31—Tb1—N11	154.06 (8)	C23—C30—H30C	109.5
N1—Tb1—N11	94.71 (8)	H30A—C30—H30C	109.5
C6—N1—C2	118.7 (3)	H30B—C30—H30C	109.5
C6—N1—Tb1	123.9 (2)	C36—N31—C32	119.8 (3)
C2—N1—Tb1	117.35 (19)	C36—N31—Tb1	122.3 (2)
N1—C2—C3	122.5 (3)	C32—N31—Tb1	117.86 (18)
N1—C2—C7	112.8 (3)	N31—C32—C33	122.2 (3)
C3—C2—C7	124.6 (3)	N31—C32—C37	114.0 (2)
C4—C3—C2	116.4 (3)	C33—C32—C37	123.7 (3)
C4—C3—C10	119.4 (3)	C34—C33—C32	116.6 (3)
C2—C3—C10	124.2 (3)	C34—C33—C40	119.6 (3)
C5—C4—C3	121.3 (3)	C32—C33—C40	123.8 (3)
C5—C4—H4	119.3	C35—C34—C33	121.4 (4)
C3—C4—H4	119.3	C35—C34—H34	119.3
C4—C5—C6	118.6 (3)	C33—C34—H34	119.3
C4—C5—H5	120.7	C34—C35—C36	118.3 (3)
C6—C5—H5	120.7	C34—C35—H35	120.9
N1—C6—C5	122.5 (3)	C36—C35—H35	120.9
N1—C6—H6	118.8	N31—C36—C35	121.7 (3)
C5—C6—H6	118.8	N31—C36—H36	119.2
O9—C7—O8	124.5 (3)	C35—C36—H36	119.2
O9—C7—C2	119.6 (3)	O39—C37—O38	124.8 (3)
O8—C7—C2	115.9 (3)	O39—C37—C32	121.1 (3)
C7—O8—Tb1	125.82 (19)	O38—C37—C32	114.1 (3)
C7—O9—Na41	172.7 (2)	C37—O38—Tb1	129.57 (19)
C3—C10—H10A	109.5	C37—O39—Na41^iv^	124.7 (2)
C3—C10—H10B	109.5	C33—C40—H40A	109.5
H10A—C10—H10B	109.5	C33—C40—H40B	109.5
C3—C10—H10C	109.5	H40A—C40—H40B	109.5
H10A—C10—H10C	109.5	C33—C40—H40C	109.5
H10B—C10—H10C	109.5	H40A—C40—H40C	109.5
C16—N11—C12	119.3 (3)	H40B—C40—H40C	109.5
C16—N11—Tb1	124.5 (2)	O42—Na41—O9	104.30 (10)
C12—N11—Tb1	116.12 (19)	O42—Na41—O39^ii^	87.81 (9)
N11—C12—C13	122.5 (3)	O9—Na41—O39^ii^	96.24 (9)
N11—C12—C17	113.6 (3)	O42—Na41—O29^i^	107.23 (11)
C13—C12—C17	123.9 (3)	O9—Na41—O29^i^	148.47 (10)
C14—C13—C12	115.4 (3)	O39^ii^—Na41—O29^i^	84.99 (9)
C14—C13—C20	120.9 (3)	O42—Na41—O43	102.01 (10)
C12—C13—C20	123.7 (3)	O9—Na41—O43	96.01 (9)
C15—C14—C13	122.5 (4)	O39^ii^—Na41—O43	161.88 (10)
C15—C14—H14	118.7	O29^i^—Na41—O43	77.59 (9)
C13—C14—H14	118.7	H42A—O42—H42B	117 (4)
C14—C15—C16	118.3 (3)	H43A—O43—H43B	107 (3)
C14—C15—H15	120.9	H44A—O44—H44B	115 (4)
C16—C15—H15	120.9	C46—C45—H45A	109.5
N11—C16—C15	121.9 (3)	C46—C45—H45B	109.5
N11—C16—H16	119	H45A—C45—H45B	109.5
C15—C16—H16	119	C46—C45—H45C	109.5
O19—C17—O18	124.6 (3)	H45A—C45—H45C	109.5
O19—C17—C12	119.8 (3)	H45B—C45—H45C	109.5
O18—C17—C12	115.5 (3)	O47—C46—C45	113.5 (4)
C17—O18—Tb1	125.3 (2)	O47—C46—H46A	108.9
C13—C20—H20A	109.5	C45—C46—H46A	108.9
C13—C20—H20B	109.5	O47—C46—H46B	108.9
H20A—C20—H20B	109.5	C45—C46—H46B	108.9
C13—C20—H20C	109.5	H46A—C46—H46B	107.7
H20A—C20—H20C	109.5	C46—O47—H47	111 (3)
H20B—C20—H20C	109.5		

Symmetry codes: (i) −*x*+2, −*y*+2, *z*−1/2; (ii) −*x*+3/2, *y*+1/2, *z*−1/2; (iii) −*x*+2, −*y*+2, *z*+1/2; (iv) −*x*+3/2, *y*−1/2, *z*+1/2.

### Hydrogen-bond geometry (Å, °)

**Table:**

*D*—H···*A*	*D*—H	H···*A*	*D*···*A*	*D*—H···*A*
O42—H42A···O47	0.81 (2)	1.95 (2)	2.742 (4)	163 (4)
O42—H42B···O38^ii^	0.81 (2)	2.01 (2)	2.819 (3)	169 (4)
O43—H43A···O28^i^	0.82 (2)	1.99 (2)	2.796 (3)	171 (4)
O43—H43A···O29^i^	0.82 (2)	2.53 (3)	3.050 (3)	122 (3)
O43—H43B···O44^i^	0.81 (2)	2.02 (2)	2.794 (4)	162 (3)
O44—H44A···O8	0.80 (4)	2.31 (4)	3.103 (4)	170 (4)
O44—H44A···O9	0.80 (4)	2.34 (4)	2.943 (4)	133 (4)
O44—H44B···O43	0.80 (4)	2.07 (4)	2.790 (4)	151 (4)
O47—H47···O19^ii^	0.80 (2)	1.96 (2)	2.726 (3)	159 (4)

Symmetry codes: (ii) −*x*+3/2, *y*+1/2, *z*−1/2; (i) −*x*+2, −*y*+2, *z*−1/2.
